# Race/skin color and educational inequalities in toothache and access to dental care among adults in Brazil: National Oral Health Survey2023

**DOI:** 10.1590/1980-549720260012.supl.1

**Published:** 2026-07-10

**Authors:** Jonatan da Rosa Pereira da Silva, Rafael Amaral Oliveira, Cleidiane Aparecida de Quadra, Afonso Valau de Lima, Gisele Silveira Coelho Lopes, Luciane Bisognin Ceretta

**Affiliations:** I Universidade Federal do Rio Grande do Sul, Department of Social Medicine – Porto Alegre (RS), Brazil.; II Universidade do Extremo Sul Catarinense, Post-graduate Program in Health Management – Santa Catarina (RS), Brazil.; III Universidade do Extremo Sul Catarinense, Socioeconomic Development and Innovation Observatory – Santa Catarina (RS), Brazil.

**Keywords:** Toothache, Racial groups, Educational status, Health status disparities, Health surveys

## Abstract

**Objective:**

To assess the association between race/skin color and educational level with toothache and access to dental care among adults aged 35–44 years in Brazil.

**Methods:**

This cross-sectional study included 8,597 adults who participated in the Brazilian National Oral Health Survey 2023 (SB 2023). Two outcomes were evaluated: the prevalence of toothache in the previous six months and access to dental care among adults who reported toothache. Crude and adjusted Poisson regression models with robust variance were used to estimate the associations of race/skin color, educational level, and the intersection of these two social markers with the outcomes.

**Results:**

The prevalence of toothache in the previous six months was 23.7%. Among adults who reported toothache, 34.1% did not access dental care. Black adults (adjusted prevalence ratio [PRa]=1.32; 95%CI 1.1–1.7; p=0.034) and those with low educational attainment (aPR=2.1; 95%CI 1.4–2.9; p<0.0001) showed a higher prevalence of toothache. In the intersectional analysis, Black adults with low educational attainment had a higher prevalence of toothache (aPR=3.5; 95%CI 2.0–6.0; p<0.0001) and of not having consulted a dentist despite experiencing pain (PRa=2.5; 95%CI 1.3–4.0; p=0.006) compared with White adults with high educational attainment.

**Conclusion:**

Black adults with low educational attainment showed a higher prevalence of toothache and lack of access to dental care even when experiencing pain, indicating that race/skin color and educational level are important determinants of oral health inequalities in Brazil.

## INTRODUCTION

The International Association for the Study of Pain (IASP) defines pain as an unpleasant sensory and emotional experience associated with actual or potential tissue damage^1^. The World Health Organization (WHO) recognizes that pain, suffering, mental health disorders, and social deprivation can result from oral diseases, producing impacts that manifest both individually and collectively^2,3^.

According to a recent systematic review with meta-analysis, the global prevalence of toothache among adults may reach 24%. However, significant geographical differences are observed, with prevalences of 43.2% in Africa, 30.6% in Asia, 17% in South America, and 15% in Oceania^4^. 4. In Brazil, studies from 2012 and 2013 indicate pain prevalences among adults ranging between 15^5^ and 21%^6^, respectively. More current statistics regarding the prevalence of toothache among adults in the country are still needed.

The literature highlights that toothache constitutes the primary reason for seeking dental care among adults worldwide^,7,8^, as it limits or prevents the performance of daily activities related to work, leisure, and interpersonal relationships^9^. Despite this, evidence indicates that the average time for individuals in pain to seek and gain access to a dentist exceeds 60 days^10^, resulting in the performance of tooth extractions in more than half of the cases^8,11^. Furthermore, studies indicate that toothache is among the leading causes of work absenteeism^12^.

In the Brazilian context, investigations analyzing toothache and access to dental care have identified relevant determinants for a lack of access, including individual aspects such as age, educational attainment, and race/skin color and structural factors such as the distance between one's residence and the referral service, insufficient availability of care, and the limited operating hours of basic health units^10–12^.

Historically, health in Brazil has been marked by highly significant inequalities, rendering the social determinants of health central to the effort to address health disparities^13^. This scenario underscores the need to understand how the intersection of various determinants of inequality, such as race and educational attainment, can lead to adverse health outcomes. Introduced into the international literature several decades ago, the concept of intersectionality can serve as an analytical tool for understanding this scenario, as it seeks to elucidate how different axes of oppression (race, class, sex, and education) intersect in shaping the health-disease process^14–16^.

In light of the foregoing, this study aimed to measure the association between race/skin color and educational attainment on the one hand, and toothache and access to dental care on the other, among adults aged 35 to 44 in Brazil.

## METHODS

### Study population, sampling and data collection

This was a cross-sectional study using data from the National Oral Health Survey (SB 2023), which assesses the oral health conditions of the Brazilian population and is conducted nationally every 10 years. The SB is part of the health surveillance component of the National Oral Health Policy (PNSB), better known as "Brasil Sorridente" (Smiling Brazil).

The SB is an epidemiological survey employing a complex sampling design, with selection carried out in one or two stages, depending on the age group. The selection stages consisted of census tracts and households, resulting in a total of 50,800 individuals interviewed within the selected households. The survey's scope encompassed 53 strata, categorized into the Federal District, state capitals, and municipalities in the interior of each state. Within these strata, clusters (census tracts) and households were randomly selected. The sample size was predetermined for both the capitals and the interior regions, taking into account the age groups surveyed in the SB 2023 (5–12 years, 15–19 years, 35–44 years, and 65–74 years).

The selection of age groups was based on criteria suggested by the World Health Organization (WHO) for oral health surveys, as well as technical considerations regarding the oral health context in Brazil. Specifically: 5 years: an age of interest for assessing the primary dentition; 12 years: an age of interest for global caries monitoring and for comparisons with international surveys; 15–19 years: an age of interest for comparison with previous SB surveys (SB 2003 and SB 2010); 35–44 years: a standard group for assessing oral health conditions in adults, the cumulative effect of caries, the severity level of periodontal involvement, and the general impact of oral health care provided within the country; and 65–74 years: a group of interest for monitoring the effects that changes in age distribution and increased life expectancy have had on the oral health of older people^17^. For this study, data from the adult age group (35–44 years; n = 9,019 individuals) were analyzed, taking into account the technical guidelines of the SB survey outlined above, as well as the fact that this age group represents a significant segment of the country's workforce.

In the interior regions, 458 census tracts located across 395 distinct municipalities were randomly selected, while in the state capitals, 1,365 census tracts were selected. It is worth noting that every age group included in the SB 2023 survey was sampled, and the data collection period spanned from March 2022 to April 15, 2024. Interviews and dental examinations were conducted by a previously trained team comprising professionals from the Unified Health System (SUS). Further details regarding the sampling methodology for the SB 2023 survey can be found in other publications^17^.

### Outcomes

Two outcomes were analyzed: toothache within the last six months, and access to a dentist among individuals who experienced a toothache during that period.

The occurrence of toothache was assessed using the question: "In the last 6 months, have you had a toothache?" The response options were "yes," "no," and "I don't know" or "I prefer not to answer." For analytical purposes, this variable was dichotomized into yes/no, and individuals who did not answer the question were excluded from the analysis (n=85).

Access to a dentist, specifically in cases where the participant reported having had a toothache, was measured using the question: "When did you last see a dentist?" The possible response options were "never been to a dentist," "within the last year," "more than one year but no more than two years ago," and "more than two years but no more than three years ago."

Adults who reported a toothache within the last six months and stated that they had seen a dentist within the last year were categorized into the group that experienced pain and had access to a dentist. All other adults who reported pain but had either never seen a dentist or had their last appointment more than one, two, or three years ago were categorized into the group that experienced pain but lacked access. For analytical purposes, the variable regarding access in the presence of pain was dichotomized into "yes" (experienced pain and had access) and "no" (experienced pain but lacked access).

### Independent variables

The independent variables considered were educational attainment and self-declared race/skin color. Information regarding educational attainment was collected via the question: "What is the highest course/grade/school year the participant attended?" The response options were: "did not attend school," "completed an adult literacy course," "incomplete elementary education," "complete elementary education," "incomplete high school education," "complete high school education," "incomplete higher education," "complete higher education," "I don't know," and "I prefer not to answer." For analytical purposes and taking into account the average educational attainment in Brazil according to data from the Brazilian Institute of Geography and Statistics (IBGE), educational attainment was categorized into three levels: high (complete or incomplete higher education), medium (complete or incomplete high school education), and low (ranging from complete elementary education to those who never attended school). Adults who did not report their educational attainment level were excluded from the analysis (n=95).

Self-declared race/skin color was collected via the question: "Regarding race/skin color, how do you identify?" The response options were: "White," "Black," "Yellow," "Brown," "Indigenous," "I don't know," and "I prefer not to answer." For the analysis, race was categorized into White and Black (combining Brown and Black). Indigenous and Asian individuals were excluded due to insufficient sample size for analysis (n=164), as were adults who did not report their race/skin color (n=78).

To investigate the effect of the intersectionality between race/skin color and educational attainment level on the outcomes, an intercategorical approach was employed to combine the response options from both questions^15^. This approach resulted in a single independent variable comprising six categories: White with high educational attainment, White with medium educational attainment, White with low educational attainment, Black with high educational attainment, Black with medium educational attainment, and Black with low educational attainment.

### Covariables

The following covariates were considered in the analysis: age (collected as a continuous variable and dichotomized for analysis into "34–39 years" and "40–44 years"), sex ("male" and "female"), city type ("capital" or "interior"), and whether the individual had private dental insurance ("yes" or "no").

### Statistical analysis

Descriptive analysis was stratified by the occurrence of toothache, and all data were presented as relative frequencies and 95% confidence intervals (95%CI). Differences between groups were assessed using the Rao-Scott χ^2^ test (Table 1).

**Table 1:** Sample characteristics according to the prevalence of toothache among adults aged 35 to 44 years in the last 6 months. National Oral Health Survey 2023, Brazil. (n=8,597).

		Toothache in the last six months		p value
		No (76.2)	Yes (23.8)	
Age				
	35–39 years	36.8 (33.4–40.3)	12.8 (10.7–15.1)	0.183
	40–44 years	39.4 (36.0–43.0)	11 (9.2–13.2)	
Sex				
	Male	26.0 (23.9–28.5)	7.8 (6.2–9.9)	0.742
	Female	50.2 (46.8–53.4)	16.0 (13.7–18.4)	
Skin color/race				
	White	34.3 (30.3–38.5)	8.6 (6.7–10.9)	0.0276
	Black	41.9 (38.5–45.3)	15.2 (13.0–17.7)	
Education level				
	High	15.9 (13.7–18.4)	8.4 (6.7–10.5)	<0.0001
	Medium	39.8 (35.7–44.0)	11.0 (9.4–12.7)	
	Low	20.5 (16.6–24.9)	4.4 (3.2–5.8)	
City type				
	Capital	18.0 (15.6–20.7)	6.2 (5.3–7.2)	0.304
	Interior	58.2 (54.2–62.0)	17.6 (14.8–20.8)	
Has dental insurance				
	Yes	65.0 (61.4–68.3)	21.0 (18.3–23.9)	0.220
	No	11.2 (9.1–13.8)	2.8 (1.9–4.2)	

Data presented as weighted relative frequency (%). p value associated with the Rao-Scott χ^2^ test.

A Poisson regression model with robust variance was used to assess the association between educational level and race/skin color with the occurrence of toothache within the past six months. For each variable, both a crude model and a model adjusted for the study covariates were constructed (Table 2). The same analytical strategy was employed to assess the influence of the intersectionality of race/skin color and educational level on the occurrence of toothache (Table 3). All results were expressed as crude (PRc) and adjusted (PRa) prevalence ratios, along with the 95%CI. Variables with p < 0.05 were considered statistically significant. The Hosmer-Lemeshow test was used to evaluate the goodness-of-fit of the constructed models^18^.

**Table 2:** Association between race/skin color and educational attainment and prevalence of toothache in the last 6 months among adults aged 35 to 44. National Oral Health Survey 2023. Brazil (n=8,597).

Independent variables		Crude model		Adjusted model**	
		PRc (95%CI)	p value*	PRa (95%CI)	p value*
Race/skin color					
	White	Ref.	-	Ref.	
	Black	1.3 (1.1–1.7)	0.032	1.3 (1.1–1.7)	0.034
Education level					
	High	Ref.	-	Ref.	-
	Medium	1.2 (0.8–1.8)	0.284	1.2 (0.8–1.7)	0.246
	Low	1.9 (1.4–2.8)	<0.0001	2.1 (1.4–2.9)	<0.0001

Ref.: reference category; PRc: crude prevalence ratio; PRa: adjusted prevalence ratio; 95%CI: 95% confidence interval.

* p value associated with the Wald test;

** adjusted for age, sex, city type, and private dental insurance. p value associated with the Hosmer-Lemeshow test: model adjusted for race/skin color: 0.2876. Model adjusted for education level: 0.3488.

**Table 3:** Association between the intersection of race/skin color and educational attainment and the prevalence of toothache in the past 6 months among adults aged 35 to 44. National Oral Health Survey 2023. Brazil (n=8.597).

Race/skin color and education level	Crude model		Adjusted model **	
	PRc (95%CI)	p value*	PRa (95%CI)	p value*
White high education level	Ref.	-	Ref.	
White medium education level	2.2 (1.3–3.5)	0.001	2.2 (1.4–3.6)	0.001
White low education level	2.6 (2.0–5.4)	<0.0001	2.7 (2.1–5.8)	<0.0001
Black high education level	2.9 (1.6–5)	0.001	2.9 (1.6–5.4)	0.001
Black medium education level	2.2 (1.3–3.7)	0.004	2.1 (1.3–3.7)	0.004
Black low education level	3.4 (2–5.9)	<0.0001	3.5 (2–6)	<0.0001

Ref.: reference category; PRc: crude prevalence ratio; PRa: adjusted prevalence ratio; 95%CI: 95% confidence interval.

* p value associated with the Wald test;

** adjusted for age, sex, city type and if has dental insurance. p value associated with the Hosmer-Lemeshow of the adjusted model: 0.3245.

All analyses were performed using the *survey data analyses* command in Stata 12 software (Stata Corporation, College Station, TX), accounting for complex sampling effects and sample weights.

### Ethical aspects

All participants signed an informed consent form prior to the interview. The SB 2023 was approved by the National Research Ethics Commission (CONEP)/National Health Council (CNS) under Opinion No. 4.823.054. All procedures followed the recommendations of CNS Resolution 466 of 2012.

#### Data Availability Statement:

The entire dataset supporting the results of this study is available upon request from the Ministry of Health at: https://www.gov.br/saude/pt-br/composicao/saps/brasil-sorridente/sb-brasil/dados.

## RESULTS

In the SB 2023 survey, 9,019 adults were interviewed. Due to missing data in the variables used (n=422), this analysis was performed with data from 8,597 adults aged 35 to 44 years. The prevalence of toothache in the last 6 months was 23.7% (n=2,235, 95%CI 20.8–26.8%). Table 1 shows that people with toothache more frequently self-identified as Black (15.2 vs. 8.6% White, p=0.0276) and with a medium or low level of education (11 and 8.4%, respectively, vs. 4.4% high education, p<0.0001). No significant differences were found in relation to age, sex, type of city, and having private dental insurance.

According to Table 2, in the crude and adjusted analysis, Black people had a higher prevalence of toothache in the last 6 months compared to White people (PRa=1.32, 95%CI 1.1–1.7, p=0.034). Similarly, people with low education also had a higher prevalence of toothache compared to those with high education (PRa=2.1, 95%CI 1.4–2.9, p<0.0001).

Table 3 describes the prevalence of toothache according to the intersection between education and race/skin color. All categories analyzed showed a higher prevalence of toothache compared to White people with high education, showing that the intersection of race/skin color and education potentiates inequalities in the occurrence of toothache. This is even higher for Black people with low education levels, who had the highest prevalence ratio in the analysis (PRa=3.5, 95%CI 2–6, p<0.0001).

Regarding adults who reported toothache in the last six months (n=2,143), 34.1% (n=818) did not have a dental appointment in the last year, therefore, they did not have access to a dentist even in case of toothache. According to Table 4, Black adults with low education levels had a higher prevalence of toothache without access to a dentist when compared to White people with high education levels (PRa=2.5, 95%CI 1.3–4, p=0.006).

**Table 4:** Association of the intersection of race/skin color and educational attainment with access to a dentist among adults aged 35 to 44 who experienced toothache in the past 6 months. National Oral Health Survey 2023. Brazil (n=2,143).

Race/skin color and education level	Crude model		Adjusted model**	
	PRc (95%CI)	p value*	PRa (95%CI)	p value*
White high education level	Ref.	-	Ref.	
White medium education level	1.9 (0.8–4.7)	0.128	2.0 (0.8–4.9)	0.133
White low education level	2.3 (0.8–6.1)	0.096	2.3 (0.9–5.9)	0.078
Black high education level	1.2 (0.4–2.9)	0.681	1.4 (0.8–3.4)	0.404
Black medium education level	1.7 (0.8–3.4)	0.122	1.6 (0.8–3.4)	0.174
Black low education level	2.6 (1.3–4.0)	0.004	2.5 (1.3–4.0)	0.006

Ref.: reference category; PRc: crude prevalence ratio; PRa: adjusted prevalence ratio; 95%CI: 95% confidence interval.

* p value associated with the Wald test;

** adjusted for age, sex, city type and if has dental insurance. p value associated with the Hosmer-Lemeshow of the adjusted model: 0.1279.

## DISCUSION

In this study, we analyze how inequalities regarding race/skin color and educational attainment impact access to oral health care in Brazil, utilizing data from a population-based survey. Our analyses advance existing knowledge by measuring not only the isolated effects of these inequalities but also the manner in which simultaneous exposure to them heightens vulnerability to poorer outcomes, specifically the occurrence of toothache and the lack of dental care even in the presence of pain. The findings presented highlight relevant elements that warrant further investigation.

The prevalence of toothache within the past six months, as identified in our study, was 23.7%, indicating that approximately one-quarter of Brazilian adults aged 35 to 44 may experience toothache. This prevalence exceeds the rates observed in national studies conducted using data from Vigitel 2009 (15.2%)^5^ and the National Oral Health Survey 2010 (21%)^6^. Furthermore, although a systematic review with meta-analysis estimates a global prevalence of 24% among adults, in South America, this statistic stood at 15%. When considering specific timeframes, the prevalence of pain within the preceding six months, as reported by that same review, was 19.5%^4^.

Although there are limitations in comparing these statistics—due to differing data collection methods and the varying timeframes regarding the occurrence of toothache (within the last month, the last three months, or the last six months)—all the cited studies investigated the occurrence of pain among economically active adults, just as our study did. Consequently, this body of evidence reveals a concerning picture regarding toothache among adults in Brazil, given that the estimated prevalence was higher than that found in the identified national surveys^5,6^ and higher than the estimate for South America^4^. This result appears particularly concerning when viewed in light of the implications of toothache already highlighted in the literature, such as reduced quality of life, work absenteeism, and physical and emotional distress^12,19,20^.

As demonstrated by other investigations^11,21,22^, our results indicated that race/skin color and educational attainment are significant determinants of the occurrence of toothache. Low educational attainment may be associated with lower health literacy, limited access to preventive information, and greater difficulty in understanding and adopting self-care practices, thereby compromising the timely utilization of dental services^19^. Education, therefore, functions as a structural marker of inequality, reflecting social integration, income, and living conditions, factors that may explain the higher prevalence of toothache among individuals with low educational attainment identified in this study^22^.

Regarding race/skin color, we observed a higher prevalence of toothache among Black individuals, even after adjustments. Previous studies indicate that Black populations residing in areas with poorer socioeconomic indicators exhibit higher rates of tooth loss and a lower prevalence of individuals free from dental caries^21^. This pattern stems from the interplay between biological factors (sanitary conditions and water fluoridation), material living conditions, and historical inequalities in access to care. Black populations are more frequently exposed to unemployment, informal employment, and precarious housing and sanitation conditions, thereby accumulating structural disadvantages that exacerbate both the risk of dental caries and the barriers to accessing dental care.^21^

The results of our analyses indicated that simultaneous exposure to health inequalities—expressed through race/skin color and educational attainment—acts synergistically, amplifying individuals' vulnerability to poorer health outcomes, such as toothache and a lack of access to dental care when in pain, as observed in the case of Black individuals with low levels of education. These findings underscore the need to understand the intersectional nature of oral health inequalities in Brazil.

The national and international scientific literature has increasingly incorporated the concept of intersectionality as an analytical tool to understand how different axes of oppression (race, class, sex, and education) intersect in shaping the health-disease process^10,16^. As studies have noted, the analysis of oral health inequities requires acknowledging that inequalities are not merely material, but also symbolic and structural, reflecting historical power relations and social exclusion. Thus, being Black and having a low level of education in Brazil entails exposure to multiple mechanisms of exclusion: discrimination in the labor market, precarious access to health services, exposure to unhealthy environments, and—frequently—experiences of institutional racism within the healthcare system itself. These mechanisms result in cumulative health deterioration, affecting not only oral health but the entirety of one's physical and psychosocial well-being^23,24^. These considerations align with the results of our analyses, which indicated that Black individuals with low educational attainment exhibit a higher prevalence of toothache and a higher prevalence of non-access to dental care, even in instances of pain.

This study presents several limitations that should be considered when interpreting the results. As this was a cross-sectional study, it was not possible to establish causal relationships between the analyzed variables, but only to indicate associations. Furthermore, the variable regarding toothache was self-reported and may be subject to recall bias or individual perceptions of pain. It is also important to highlight that, although we included key structural markers of inequality, such as race/skin color and educational attainment, other socioeconomic and contextual dimensions that influence oral health, such as income, housing conditions, geographic access to services, and the availability of healthcare professionals, were not explored in this study. Nevertheless, the use of a population-based national survey, featuring standardized methodology and high representativeness, reinforces the robustness and relevance of the findings presented herein.

The findings of this study carry significant implications for public policy planning. First, they underscore the need for intersectoral actions aimed at reducing structural inequalities through educational policies, income redistribution, the expansion of basic sanitation, and the promotion of racial equity. Within the SUS (Brazil's Unified Health System), it is crucial to expand the reach and problem-solving capacity of oral health teams within primary health care, with a particular emphasis on oral health prevention initiatives and the timely management of pain, thereby preventing work absenteeism or future unnecessary tooth extractions. Moreover, health education strategies must take into account cultural, territorial, and linguistic specificities, thereby contributing to the enhancement of oral health literacy and the empowerment of historically marginalized populations.
