# Supplementary material for: Race/skin color and educational inequalities in toothache and access to dental care among adults in Brazil: National Oral Health Survey2023
Source: Rev Bras Epidemiol. 2026 Jul 10;29(Suppl 1):e260012supl1. doi: 10.1590/1980-549720260012.supl.1 (PMC13360981; doi:10.1590/1980-549720260012.supl.1)
Supplement: Translation

# Translation: Desigualdades de raça/cor de pele e escolaridade na dor de dente e acesso ao dentista entre adultos de 35 a 44 anos no Brasil

**DOI:** 10.1590/1980-549720260012.supl.1.2

**Authors:** Jonatan da Rosa Pereira da Silva, Rafael Amaral Oliveira, Cleidiane Aparecida de Quadra, Afonso Valau de Lima, Gisele Silveira Coelho Lopes, Luciane Bisognin Ceretta

**Affiliations:** I Universidade Federal do Rio Grande do Sul, Departamento de Medicina Social – Porto Alegre (RS), Brasil.; II Universidade do Extremo Sul Catarinense, Programa de Pós-Graduação de Gestão em Saúde – Santa Catarina (RS), Brasil.; III Universidade do Extremo Sul Catarinense, Observatório de Desenvolvimento Socioeconômico e Inovação – Santa Catarina (RS), Brasil.

**Keywords:** Odontalgia, Grupos raciais, Escolaridade, Disparidades nos níveis de saúde, Inquéritos epidemiológicos

## Abstract

**Objetivo:**

mensurar a associação da raça/cor de pele e do nível de escolaridade com a dor de dente e o acesso ao dentista entre adultos de 35-44 anos no Brasil.

**Métodos:**

estudo transversal com 8.597 adultos que participaram da Pesquisa Nacional de Saúde Bucal 2023 (SB 2023). Foram mensurados dois desfechos: a prevalência de dor de dente entre os adultos nos últimos 6 meses e o acesso ao dentista entre os adultos que tiveram dor de dente. Modelos de regressão de Poisson, com variância robusta, brutos e ajustados, foram utilizados para mensurar a associação da raça/cor de pele, da escolaridade e da intersecção desses dois marcadores com os desfechos analisados.

**Resultados:**

a prevalência de dor de dente entre os adultos nos últimos 6 meses foi de 23,7%. Entre esses adultos, 34,1% não tiveram acesso ao dentista. Os adultos negros (RPa=1,32, IC95% 1,1–1,7, p=0,034) e os com baixa escolaridade (RPa=2,1, IC95% 1,4–2,9, p<0,0001) apresentaram maior prevalência de dor de dente. Quanto à interseccionalidade, adultos negros com baixo nível de escolaridade apresentaram uma prevalência maior de dor de dente (RPa=3,5, IC95% 2,0–6,0, p<0,0001) e de não terem consultado um dentista mesmo em caso de dor (RPa=2,5, IC95% 1,3–4,0, p=0,006) comparados aos adultos brancos com alto nível de escolaridade.

**Conclusão:**

adultos negros com baixa escolaridade apresentaram uma maior prevalência de dor de dente e de não terem consultado um dentista mesmo em caso de dor, indicando que a raça/cor de pele e o nível de escolaridade são importantes determinantes para a saúde bucal no Brasil.

## INTRODUÇÃO

A International Association for the Study of Pain (IASP) define dor como uma experiência sensorial e emocional desagradável, associada a dano real ou potencial aos tecidos^1^. A Organização Mundial da Saúde (OMS) reconhece que a dor, o sofrimento, os transtornos de saúde mental e a privação social podem resultar de doenças bucais, produzindo impactos que se manifestam tanto individual quanto coletivamente^2,3^.

De acordo com uma recente revisão sistemática com metanálise, a prevalência mundial de dor de dente entre adultos pode alcançar 24%. Contudo, observam-se diferenças geográficas importantes, com prevalências de 43,2% na África, 30,6% na Ásia, 17% na América do Sul e 15% na Oceania^4^. No Brasil, estudos de 2012 e 2013 apontam prevalências de dor entre adultos que variam entre 15^5^ e 21%^6^, respectivamente. Estatísticas mais atuais sobre a prevalência de dor de dente entre adultos no país ainda são necessárias.

A literatura destaca que a dor de dente constitui o principal motivo de procura por atendimento odontológico entre adultos no mundo^7,8^, pois limita ou impede a realização de atividades cotidianas relacionadas ao trabalho, ao lazer e às relações interpessoais^9^. Apesar disso, evidências indicam que o tempo médio para que indivíduos com dor procurem e tenham acesso a um dentista ultrapassa 60 dias^10^, resultando, em mais da metade dos casos, na realização de extrações dentárias^8,11^. Além disso, estudos apontam que a dor de dente está entre as principais causas de absenteísmo laboral^12^.

No contexto brasileiro, investigações que analisaram a dor de dente e o acesso ao dentista identificaram determinantes relevantes para a falta de acesso, incluindo aspectos individuais, como idade, nível de escolaridade e raça/cor de pele, e fatores estruturais, como distância entre a residência e o serviço de referência, a disponibilidade insuficiente de atendimento e o horário reduzido de funcionamento das unidades básicas de saúde^10–12^.

A saúde no Brasil, historicamente, é marcada por desigualdades bastante expressivas, tornando os determinantes sociais de saúde protagonistas no enfretamento de desigualdades em saúde^13^. Esse cenário reflete a necessidade de entender como a sobreposição a diferentes determinantes de desigualdades, como a raça e o nível de escolaridade, pode produzir desfechos desfavoráveis à saúde. Introduzido na literatura internacional há algumas décadas, o conceito da interseccionalidade pode ser utilizado como ferramenta analítica para compreender esse cenário, uma vez que se propõe a entender como diferentes eixos de opressão (raça, classe, gênero, escolaridade) se entrecruzam na determinação do processo saúde-doença^14–16^.

Diante do exposto, este estudo teve como objetivo mensurar a associação da raça/cor de pele e do nível de escolaridade com a dor de dente e o acesso ao dentista entre adultos de 35 a 44 anos no Brasil.

## MÉTODOS

### População do estudo, amostragem e coleta de dados

Trata-se de um estudo transversal que utilizou dados da Pesquisa Nacional de Saúde Bucal (SB 2023), que trata das condições de saúde bucal da população brasileira e que ocorre a cada 10 anos, em nível nacional. A SB faz parte do componente de vigilância à saúde da Política Nacional de Saúde Bucal (PNSB), mais conhecida como Brasil Sorridente.

A SB é um levantamento epidemiológico com amostra complexa, selecionada em um ou dois estágios, dependendo do grupo etário. Os estágios de seleção foram setor censitário e domicílio, alcançando um total de 50.800 pessoas entrevistadas nos domicílios sorteados. A abrangência da pesquisa foi de 53 estratos, divididos em Distrito Federal, capitais e municípios do interior de cada estado. Nos estratos foram sorteados conglomerados (setores censitários) e domicílios. O tamanho da amostra foi pré-definido para capitais e para o interior, considerando os grupos etários pesquisados na SB 2023 (5–12 anos, 15–19 anos, 35–44 anos e 65 a 74 anos).

A seleção dos grupos etários foi baseada em aspectos sugeridos pela Organização Mundial da Saúde (OMS) para levantamentos da saúde bucal e considerações técnicas sobre o contexto de saúde bucal no Brasil. A saber: 5 anos: idade de interesse para avaliação da dentição decídua; 12 anos: idade de interesse para monitoramento global da cárie e comparações com inquéritos internacionais; 15–19 anos: idade de interesse para comparação com pesquisas anteriores da SB (SB 2003 e SB 2010); 35–44 anos: grupo padrão para avaliação das condições de saúde bucal em adultos, efeito total da cárie, de nível de gravidade do envolvimento periodontal e efeitos gerais da assistência à saúde bucal prestada no país; e 65–74 anos: grupo de interesse para monitorar os efeitos que as mudanças na distribuição etária e o aumento da expectativa de vida têm produzido na saúde bucal dos idosos^17^. Para o estudo, foram analisados dados do grupo etário adultos (35–44 anos, n=9.019 indivíduos), considerando as indicações técnicas da SB apresentadas e o fato de esse grupo etário representar uma parcela importante do mercado de trabalho do país.

No interior, foram sorteados 458 setores censitários, localizados em 395 municípios distintos, e nas capitais foram sorteados 1.365 setores censitários. Cabe destacar que cada grupo etário da SB 2023 foi selecionado e que o período de coleta de dados foi de março de 2022 a 15 de abril de 2024. As entrevistas e os exames odontológicos foram realizados por equipe previamente treinada, formada por profissionais do Sistema Único de Saúde (SUS). Mais detalhes sobre a amostragem da SB 2023 podem ser consultados em outras publicações^17^.

### Desfechos

Foram analisados dois desfechos: dor de dente nos últimos seis meses e acesso ao dentista entre pessoas que tiveram dor de dente nos últimos seis meses.

A ocorrência de dor de dente foi avaliada por meio da pergunta: "Nos últimos 6 meses, você teve dor de dente?". As opções de resposta eram "sim", "não" e "não sei" ou "não quero responder". Para fins de análise, essa variável foi dicotomizada em sim/não, e pessoas que não responderam à pergunta foram excluídas da análise (n=85).

O acesso ao dentista nos casos em que o participante declarou que teve dor de dente foi mensurado por meio da pergunta "Quando você consultou com o dentista pela última vez?". As possibilidades de resposta eram "nunca foi ao dentista", "até um ano", "mais de um ano a dois anos" e "mais de dois anos a três anos".

Adultos que relataram dor de dente nos últimos seis meses e que afirmaram que consultaram um dentista até um ano foram categorizados no grupo que teve dor e teve acesso ao dentista. Todos os demais adultos que relataram dor, mas nunca consultaram o dentista ou tiveram consulta há mais de um ano, dois anos ou três anos, foram categorizados no grupo que teve dor e não teve acesso. Para propósitos de análise, a variável de acesso em caso de dor foi dicotomizada em "sim" (teve dor e acesso) e "não" (teve dor e não teve acesso).

### Variáveis independentes

As variáveis independentes consideradas foram nível de escolaridade e raça/cor de pele autodeclarada. A informação sobre escolaridade foi coletada pela pergunta: "Qual curso/série/ano escolar mais elevado que o participante frequentou na escola?". As possibilidades de resposta eram "não estudou na escola", "fez curso de alfabetização de adultos", "ensino fundamental incompleto", "ensino fundamental completo", "ensino médio incompleto", "ensino médio completo", "ensino superior incompleto", "ensino superior completo", "não sei" e "não quero responder". Para fins de análise e considerando a escolaridade média no Brasil conforme dados do Instituto Brasileiro de Geografia e Estatística (IBGE), a escolaridade foi categorizada em três níveis: alta (ensino superior completo ou incompleto), média (ensino médio completo ou incompleto) e baixa (de ensino fundamental completo até quem nunca estudou na escola). Adultos que não informaram seu nível de escolaridade foram excluídos da análise (n=95).

A raça/cor de pele autodeclarada foi coletada pela pergunta "Quanto à raça/cor de pele, como você se declara?". As possibilidades de resposta eram "branco", "preto", "amarelo", "pardo", "indígena", "não sabe" e "não quer responder". Para a análise, a raça foi categorizada em branco e negro (pardo e preto). Foram excluídos indígenas e amarelos por falta de tamanho amostral para análise (n=164) e adultos que não informaram sua raça/cor de pele (n=78).

Para investigar o efeito da interseccionalidade de raça/cor de pele e nível de escolaridade nos desfechos, foi utilizada uma abordagem intercategórica para combinar as possibilidades de resposta de ambas as perguntas^15^. Essa abordagem resultou em uma variável independente com seis categorias: branco alta escolaridade, branco média escolaridade, branco baixa escolaridade, negro alta escolaridade, negro média escolaridade e negro baixa escolaridade.

### Covariáveis

As seguintes covariáveis foram consideradas na análise: idade (coletada de forma contínua e para análise dicotomizada em "34–39 anos" e "40–44 anos"), sexo ("masculino" e "feminino"), tipo de cidade ("capital" ou "interior") e se possuía plano odontológico privado ("sim" ou "não).

### Análise estatística

A análise descritiva foi estratificada pela ocorrência de dor de dente, e todos os dados foram apresentados em frequência relativa e intervalo de confiança de 95% (IC95%). Diferenças entre os grupos foram mensuradas pelo teste Qui-quadrado de Rao-Scott (Tabela 1).

**Tabela 1:** Características da amostra de acordo com a prevalência de dor de dente entre os adultos de 35–44 anos nos últimos 6 meses. Pesquisa de Saúde Bucal 2023, Brasil (n=8.597).

		Dor de dente nos últimos seis meses		p-valor
		Não (76,2)	Sim (23,8)	
Idade				
	35–39 anos	36,8 (33,4–40,3)	12,8 (10,7–15,1)	0,183
	40–44 anos	39,4 (36,0–43,0)	11 (9,2–13,2)	
Sexo				
	Masculino	26,0 (23,9–28,5)	7,8 (6,2–9,9)	0,742
	Feminino	50,2 (46,8–53,4)	16,0 (13,7–18,4)	
Cor de pele/raça				
	Branco	34,3 (30,3–38,5)	8,6 (6,7–10,9)	0,0276
	Negro	41,9 (38,5–45,3)	15,2 (13,0–17,7)	
Nível de escolaridade				
	Alta	15,9 (13,7–18,4)	8,4 (6,7–10,5)	<0,0001
	Médio	39,8 (35,7–44,0)	11,0 (9,4–12,7)	
	Baixo	20,5 (16,6–24,9)	4,4 (3,2–5,8)	
Tipo de cidade				
	Capital	18,0 (15,6–20,7)	6,2 (5,3–7,2)	0,304
	Interior	58,2 (54,2–62,0)	17,6 (14,8–20,8)	
Tem plano odontológico				
	Sim	65,0 (61,4–68,3)	21,0 (18,3–23,9)	0,220
	Não	11,2 (9,1–13,8)	2,8 (1,9–4,2)	

Dados apresentados por frequência relativa ponderada (%). Valor de p associado ao teste Qui-quadrado de Rao-Scott.

Um modelo de regressão de Poisson com variância robusta foi utilizado para mensurar a associação do nível de escolaridade e da raça/cor de pele com a ocorrência de dor de dente nos últimos seis meses. Para cada variável foram construídos um modelo bruto e um modelo ajustado pelas covariáveis do estudo (Tabela 2). A mesma estratégia de análise foi utilizada para mensurar a influência da interseccionalidade de raça/cor de pele e escolaridade na ocorrência de dor de dente (Tabela 3). Todos os resultados foram expressos por razões de prevalência brutas (RPb) e ajustadas (RPa) e IC95%. Foram consideradas estatisticamente significativas as variáveis com p<0,05. O teste de Hosmer e Lemeshow foi utilizado para avaliar a qualidade dos modelos criados^18^.

**Tabela 2:** Associação entre raça/cor de pele e nível de escolaridade e prevalência de dor de dente nos últimos 6 meses entre adultos de 35 a 44 anos. Pesquisa Nacional de Saúde Bucal 2023. Brasil (n=8.597).

Variáveis independentes		Modelo bruto		Modelo ajustado**	
		RPb (IC95%)	p-valor*	RPa (IC95%)	p-valor*
Raça/cor de pele					
	Branco	Ref.	-	Ref.	
	Negro	1,3 (1,1–1,7)	0,032	1,3 (1,1–1,7)	0,034
Nível de escolaridade					
	Alto	Ref.	-	Ref.	-
	Médio	1,2 (0,8–1,8)	0,284	1,2 (0,8–1,7)	0,246
	Baixo	1,9 (1,4–2,8)	<0,0001	2,1 (1,4–2,9)	<0,0001

Ref.: categoria de referência; RPb: razão de prevalência bruta; RPa: razão de prevalência ajustada; IC95%: intervalo com 95% de confiança.

* Valor de p associado ao teste de Wald;

** ajustado por idade, sexo, tipo de cidade e plano odontológico privado. Valor de p associado ao teste de Hosmer e Lemeshow: modelo ajustado de raça/cor de pele: 0,2876. Modelo ajustado de nível de escolaridade: 0,3488.

**Tabela 3:** Associação da intersecção de raça/cor de pele e escolaridade e da prevalência de dor de dente nos últimos 6 meses entre adultos de 35 a 44 anos. Pesquisa Nacional de Saúde Bucal 2023. Brasil (n=8.597).

Raça/cor de pele e escolaridade	Modelo bruto		Modelo ajustado**	
	RPb (IC95%)	p-valor*	RPa (IC95%)	p-valor*
Branco alta escolaridade	Ref.	-	Ref.	
Branco média escolaridade	2,2 (1,3–3,5)	0,001	2,2 (1,4–3,6)	0,001
Branco baixa escolaridade	2,6 (2,0–5,4)	<0,0001	2,7 (2,1–5,8)	<0,0001
Negro alta escolaridade	2,9 (1,6–5)	0,001	2,9 (1,6–5,4)	0,001
Negro média escolaridade	2,2 (1,3–3,7)	0,004	2,1 (1,3–3,7)	0,004
Negro baixa escolaridade	3,4 (2–5,9)	<0,0001	3,5 (2–6)	<0,0001

Ref.: categoria de referência; RPb: razão de prevalências bruta; RPa: razão de prevalência ajustada; IC95%: intervalo com 95% de confiança.

* Valor de p associado ao teste de Wald;

** ajustado por idade, sexo, tipo de cidade e se tem plano odontológico. Valor de p associado ao teste de Hosmer e Lemeshow do modelo ajustado: 0,3245.

Todas as análises foram realizadas usando o comando *survey data analyses*, do *software* Stata 12 (Stata Corporation, College Station, TX), e consideraram efeitos da amostragem complexa e os pesos amostrais.

### Aspectos éticos

Todos os participantes assinaram o Termo de Consentimento Livre e Esclarecido (TCLE) antes da entrevista. A SB 2023 foi aprovada pela Comissão Nacional de Ética em Pesquisa (CONEP)/Conselho Nacional de Saúde (CNS) sob o parecer n° 4.823.054. Todos os procedimentos seguiram as recomendações da Resolução 466, de 2012, do CNS.

#### Declaração de disponibilidade de dados:

Todo o conjunto de dados que dá suporte aos resultados deste estudo está disponível mediante solicitação ao Ministério da Saúde, em: https://www.gov.br/saude/pt-br/composicao/saps/brasil-sorridente/sb-brasil/dados.

## RESULTADOS

Na SB 2023, foram entrevistados 9.019 adultos. Devido a *missings* nas variáveis utilizadas (n=422), esta análise foi realizada com dados de 8.597 adultos de 35 a 44 anos. A prevalência de dor de dente nos últimos 6 meses foi de 23,7% (n=2.235, IC95% 20,8–26,8%). A Tabela 1 mostra que pessoas com dor de dente com mais frequência se autodeclaram negras (15,2% *vs*. 8,6% brancos, p=0,0276) e com nível de escolaridade médio ou baixo (11 e 8,4%, respectivamente, *vs*. 4,4% alta escolaridade, p<0,0001). Não foram encontradas diferenças significativas em relação a idade, sexo, tipo de cidade e possuir plano odontológico privado.

De acordo com a Tabela 2, na análise bruta e ajustada, pessoas negras apresentaram uma prevalência de dor de dente nos últimos 6 meses maior em comparação às pessoas brancas (RPa=1,32, IC95% 1,1–1,7, p=0,034). Da mesma forma, pessoas com baixa escolaridade também apresentaram uma prevalência maior de dor de dente comparadas às com alta escolaridade (RPa=2,1, IC95% 1,4–2,9, p<0,0001).

A Tabela 3 descreve a prevalência de dor de dente de acordo com a intersecção entre escolaridade e raça/cor de pele. Todas as categorias analisadas apresentaram uma prevalência maior de dor de dente em comparação às pessoas brancas com alta escolaridade, evidenciando que a intersecção de raça/cor de pele e escolaridade potencializa as desigualdades na ocorrência de dor de dente. Isso é ainda maior para pessoas negras com baixa escolaridade que apresentaram a maior razão de prevalência da análise (RPa=3,5, IC95% 2–6, p<0,0001).

Em relação aos adultos que relataram dor de dente nos últimos seis meses (n=2.143), 34,1% (n=818) não tiveram uma consulta odontológica no último ano, portanto, não tiveram acesso ao dentista mesmo em caso de dor de dente. De acordo com a Tabela 4, adultos negros com baixa escolaridade apresentaram uma prevalência maior de dor de dente sem acesso ao dentista quando comparados às pessoas brancas com alta escolaridade (RPa=2,5, IC95% 1,3–4, p=0,006).

**Tabela 4:** Associação da intersecção de raça/cor de pele e escolaridade com acesso ao dentista entre adultos de 35 a 44 anos que tiveram dor de dente nos últimos 6 meses. Pesquisa Nacional de Saúde Bucal 2023. Brasil (n=2.143).

Raça/cor de pele e escolaridade	Modelo bruto		Modelo ajustado**	
	RPb (IC95%)	p-valor*	RPa (IC95%)	p-valor*
Branco alta escolaridade	Ref.	-	Ref.	
Branco média escolaridade	1,9 (0,8–4,7)	0,128	2,0 (0,8–4,9)	0,133
Branco baixa escolaridade	2,3 (0,8–6,1)	0,096	2,3 (0,9–5,9)	0,078
Negro alta escolaridade	1,2 (0,4–2,9)	0,681	1,4 (0,8–3,4)	0,404
Negro média escolaridade	1,7 (0,8–3,4)	0,122	1,6 (0,8–3,4)	0,174
Negro baixa escolaridade	2,6 (1,3–4,0)	0,004	2,5 (1,3–4,0)	0,006

Ref.: categoria de referência; RPb: razão de prevalências bruta; RPa: razão de prevalência ajustada; IC95%: intervalo com 95% de confiança.

* Valor de p associado ao teste de Wald;

** ajustado por idade, sexo, tipo de cidade e se tem plano odontológico. Valor de p associado ao teste de Hosmer e Lemeshow do modelo ajustado: 0,1279.

## DISCUSSÃO

Neste estudo, analisamos como desigualdades de raça/cor de pele e escolaridade impactam o acesso à saúde bucal no Brasil, utilizando dados de uma pesquisa de base populacional. Nossas análises avançam o conhecimento existente ao mensurar não apenas os efeitos isolados dessas desigualdades, mas também a forma como sua exposição simultânea potencializa a vulnerabilidade a piores desfechos, como a ocorrência de dor de dente e a ausência de atendimento odontológico mesmo diante da dor. Os achados apresentados evidenciam elementos relevantes que merecem aprofundamento.

A prevalência de dor de dente nos últimos seis meses, encontrada em nosso estudo, foi de 23,7%, indicando que cerca de um quarto dos adultos brasileiros de 35 a 44 anos pode experimentar dor de dente. Tal prevalência é superior às observadas em estudos nacionais realizados com dados do Vigitel 2009 (15,2%)^5^ e da Pesquisa Nacional de Saúde Bucal 2010 (21%)^6^. Além disso, embora uma revisão sistemática com metanálise estime prevalência global de 24% entre adultos, na América do Sul essa estatística foi de 15%. Ao considerar recortes temporais, a prevalência de dor nos últimos seis meses encontrada por essa revisão foi de 19,5%^4^.

Embora existam limitações na comparação dessas estatísticas, devido às diferentes metodologias de coleta dos dados e da temporalidade da ocorrência da dor de dente (no último mês, nos últimos três ou nos últimos seis meses), todos os estudos citados investigaram a ocorrência de dor entre adultos economicamente ativos, assim como em nossa pesquisa. Logo, o conjunto dessas evidências revela um cenário preocupante para a dor de dente entre adultos no Brasil, uma vez que a prevalência estimada foi maior do que a das pesquisas nacionais identificadas^5,6^ e do que a estimada para a América do Sul^4^. Esse resultado também se mostra preocupante quando refletido à luz das implicações da dor de dente já indicadas pela literatura, como a redução da qualidade de vida, o absenteísmo laboral e o sofrimento físico e emocional^12,19,20^.

Assim como demonstrado por outras investigações^11,21,22^, nossos resultados indicaram que a raça/cor de pele e o nível de escolaridade são determinantes relevantes para a ocorrência de dor de dente. O baixo nível de escolaridade pode se relacionar a menor literacia em saúde, a acesso limitado a informações de prevenção e a maior dificuldade de compreensão e adoção de práticas de autocuidado, comprometendo o uso oportuno dos serviços odontológicos^19^. A escolaridade, portanto, funciona como marcador estrutural de desigualdades, refletindo inserção social, renda e condições de vida, o que pode explicar a maior prevalência de dor de dente entre pessoas com baixa escolaridade identificada neste estudo^22^.

No que se refere à raça/cor de pele, observamos maior prevalência de dor de dente entre pessoas negras, mesmo após ajustes. Estudos prévios mostram que populações negras residentes em áreas com piores indicadores socioeconômicos apresentam maior perda dentária e menor prevalência de indivíduos livres de cárie^21^. Tal padrão decorre da interação entre fatores biológicos (condições sanitárias e fluoretação da água), condições materiais de vida e desigualdades históricas de acesso ao cuidado. As populações negras estão mais frequentemente expostas ao desemprego, à informalidade e à precarização das condições de moradia e saneamento, acumulando desvantagens estruturais que ampliam tanto o risco à cárie quanto barreiras ao atendimento odontológico^21^.

Os resultados de nossas análises indicaram que a exposição simultânea a desigualdades em saúde, expressa pela raça/cor de pele e pelo nível de escolaridade, atua de forma sinérgica, potencializando a vulnerabilidade das pessoas a piores desfechos em saúde, como a dor de dente e a falta de acesso ao dentista em situação de dor, como no caso de pessoas negras com baixo nível de escolaridade. Esses achados reforçam a necessidade de entender o caráter interseccional das desigualdades em saúde bucal no Brasil.

A literatura científica nacional e internacional vem incorporando o conceito da interseccionalidade como ferramenta analítica para compreender como diferentes eixos de opressão (raça, classe, gênero, escolaridade) se entrecruzam na determinação do processo saúde-doença^10,16^. Conforme mencionam estudos, a análise das iniquidades em saúde bucal requer reconhecer que as desigualdades não são apenas materiais, mas também simbólicas e estruturais, expressando relações históricas de poder e exclusão social. Assim, ser negro e ter baixa escolaridade no Brasil significa estar exposto a múltiplos mecanismos de exclusão: discriminação no mercado de trabalho, precarização do acesso aos serviços de saúde, exposição a ambientes insalubres e, muitas vezes, experiências de racismo institucional no próprio sistema de saúde. Esses mecanismos resultam em adoecimento cumulativo, afetando não apenas a saúde bucal, mas a integralidade do bem-estar físico e psicossocial^23,24^. Tais considerações vão ao encontro dos resultados de nossas análises, que indicaram que pessoas negras com baixa escolaridade têm maior prevalência de dor de dente e maior prevalência de não acesso ao dentista mesmo em casos de dor.

Este estudo apresenta algumas limitações que devem ser consideradas na interpretação dos resultados. Por se tratar de um estudo transversal, não é possível estabelecer relações de causalidade entre as variáveis analisadas, apenas indicar associações. Ademais, a variável dor de dente foi autorreferida e pode estar sujeita ao viés de memória ou percepção individual da dor. Também é importante destacar que, embora tenhamos incluído importantes marcadores estruturais de desigualdade, como raça/cor de pele e nível de escolaridade, outras dimensões socioeconômicas e contextuais que influenciam a saúde bucal, como renda, condições de moradia, acesso geográfico aos serviços e disponibilidade de profissionais, não foram exploradas neste estudo. Ainda assim, o uso de um inquérito nacional de base populacional, com metodologia padronizada e alta representatividade, fortalece a robustez e a relevância dos achados apresentados.

Os achados deste estudo possuem importantes implicações para o planejamento de políticas públicas. Inicialmente, ressaltam a necessidade de ações intersetoriais voltadas à redução das desigualdades estruturais, por meio de políticas educacionais, de redistribuição de renda, de ampliação do saneamento básico e de promoção da equidade racial. No SUS, torna-se fundamental ampliar o alcance e a resolutividade das equipes de saúde bucal na Atenção Primária à Saúde (APS), com ênfase nas ações de prevenção em saúde bucal e no atendimento oportuno da dor, evitando o absenteísmo laboral ou futuras extrações desnecessárias. Ademais, estratégias de educação em saúde devem considerar especificidades culturais, territoriais e linguísticas, contribuindo para o fortalecimento da literacia em saúde bucal e para o empoderamento das populações historicamente marginalizadas.
